# Food and agricultural wastes-derived biochars in combination with mineral fertilizer as sustainable soil amendments to enhance soil microbiological activity, nutrient cycling and crop production

**DOI:** 10.3389/fpls.2022.1028101

**Published:** 2022-10-06

**Authors:** Adnan Mustafa, Martin Brtnicky, Tereza Hammerschmiedt, Jiri Kucerik, Antonin Kintl, Tomas Chorazy, Muhammad Naveed, Petr Skarpa, Tivadar Baltazar, Ondrej Malicek, Jiri Holatko

**Affiliations:** ^1^ Department of Agrochemistry, Soil Science, Microbiology and Plant Nutrition, Faculty of AgriSciences, Mendel University in Brno, Brno, Czechia; ^2^ Institute of Chemistry and Technology of Environmental Protection, Faculty of Chemistry, Brno University of Technology, Brno, Czechia; ^3^ Institute for Environmental Studies, Faculty of Science, Charles University in Prague, Praha, Czechia; ^4^ Agricultural Research, Ltd., Troubsko, Czechia; ^5^ AdMaS Research Centre, Faculty of Civil Engineering, Brno University of Technology, Brno, Czechia; ^6^ Institute of Soil and Environmental Science, University of Agriculture Faisalabad, Faisalabad, Pakistan; ^7^ Agrovyzkum Rapotin, Ltd., Rapotin, Czechia

**Keywords:** nutrient cycling, sustainable crop production, waste recycling, food security, agriculture

## Abstract

The ever-increasing human population associated with high rate of waste generation may pose serious threats to soil ecosystem. Nevertheless, conversion of agricultural and food wastes to biochar has been shown as a beneficial approach in sustainable soil management. However, our understanding on how integration of biochar obtained from different wastes and mineral fertilizers impact soil microbiological indicators is limited. Therefore, in the present study the effects of agricultural (AB) and food waste derived (FWB) biochars with and without mineral fertilizer (MF) on crop growth and soil health indicators were compared in a pot experiment. In particular, the impacts of applied amendments on soil microbiological health indicators those related to microbial extracellular (C, N and P acquiring) enzymes, soil basal as well as different substrate induced respirations along with crop’s agronomic performance were explored. The results showed that compared to the control, the amendment with AB combined with MF enhanced the crop growth as revealed by higher above and below ground biomass accumulation. Moreover, both the biochars (FWB and AB) modified soil chemical properties (pH and electric conductivity) in the presence or absence of MF as compared to control. However, with the sole application of MF was most influential strategy to improve soil basal and arginin-induced respiration as well as most of the soil extracellular enzymes, those related to C, N and P cycling. Use of FWB resulted in enhanced urease activity. This suggested the role of MF and FWB in nutrient cycling and plant nutrition. Thus, integration of biochar and mineral fertilizers is recommended as an efficient and climate smart package for sustainable soil management and crop production.

## Introduction

The world’s population has been increasing exponentially and is expected to turn up to 9.6 billion until 2050 (Tripathi et al., 2019) and is projected to be linked with a 60% increase in food demand (Boretti and Rosa, 2019). This overwhelming pace of human population, high food consumption and agricultural waste production will put pressure on the global agriculture which may outcome in negative environmental and socio-economic aspects. In fact, the higher food production and waste generation due to human consumption are concomitantly linked and approximately 1/3^rd^ of the food produced is annually wasted around the globe (Kibler et al., 2018; Ishangulyyev et al., 2019). It has been estimated that the annual amount of this food waste is approximately 1.3 billion tons globally (Gustavsson et al., 2011). A big part of this amount (56%) is produced by developed world while the rest (44%) is being generated by the less developed countries (Bond et al., 2013; Lipinski et al., 2013). However, a big part of the wasted food material is lost, incinerated or buried in the landfills, causing soil and water pollution which is another of the main global concerns (Parry et al., 2007; Abiad and Meho, 2018). In this way, only in USA, around US$90 billion–US$100 billion a year is lost (Lundqvist et al., 2008). Therefore, the situation demands for the safer utilization of food and viable approaches to deal with the wasted materials to ensure the food security and environmental protection.

Several types of organic (including food and agricultural) wastes are generated worldwide with the potential to be utilized as soil amendments for enhancing soil health and crop production (Toscano et al., 2013; Sayara et al., 2020; Naveed et al., 2021). However, direct application of such wastes may cause risks to soil health, especially to soil chemical and microbiological characters (Urra et al., 2019). Therefore, bioconversion of agricultural and food wastes to non-hazardous and stable soil amendments is a viable alternative. This will not only reduce the risks associated with environmental burdens, but also ensures the safe disposal and utilization of end product as sustainable soil amendments (Sulok et al., 2021; Brtnicky et al., 2022). Conversion of agricultural and food wastes into biochar (a C rich) product produced by the pyrolysis is an effective way in this regard too. Biochar has been reported to enhance soil fertility, improve soil health and ultimately increasing crop yields (Ahmad et al., 2020; Karimi et al., 2020; Rasool et al., 2022). We took advantage of converting the collected food and agricultural wastes into biochars and utilized them for this study.

It has been recognized that intensive agricultural practices, injudicious use of chemical fertilizers, removal of crop straw and heavy tillage operations have resulted in the loss of soil fertility and degradation of arable lands (Sonmez et al., 2016). Currently, farmers heavily rely on the use of chemical fertilizers and crop protection chemicals to produce higher crop yields (Mustafa et al., 2019). This behavior of farmers has aggravates the soil degradation and its productive capacity as the higher use of chemicals and fertilizers deteriorate the environmental resources and cause soil salinity, eutrophication and heavy metal pollution in arable soils (Bouraoui and Grizzetti, 2014; Ali et al., 2019; Zulfiqar et al., 2022). To entail these challenges, researchers are focusing on developing alternative strategies, which ensure high crop yields without negative effects on the soil quality and water resources (Bais-Moleman et al., 2019; Tan et al., 2021; Kang et al., 2022; Wali et al., 2022). Nevertheless, chemical fertilizers have shown a potential to increase crop yields by modifying soil properties, the sole utilization of chemical fertilizers have been questioned in the face of climate change (Srivastav, 2020; Meena et al., 2020). In this respect, the combination of biochar together with mineral fertilizers could be an effective strategy to enhance soil health and crop biomass yields while keeping the mineral fertilizers at low levels. Previously many studies have shown the improvements in crop yields and soil fertility under the application of either chemical fertilizers or biochar (Atkinson et al., 2010; Khadem and Raiesi, 2017) or the combination of both (Singh et al., 2019a; Singh et al., 2019b). Most of these studies have shown the variable effects of biochar derived from various sources on soil properties (Prendergast-Miller et al., 2014; Hussain et al., 2017; Mohan et al., 2018) and agronomic and physiological responses of crops (Carter et al., 2013; Kuppusamy et al., 2016; Singh et al., 2020). Majority of these studies have only focused on soil physico-chemical properties and the role of applied biochar amendments on soil microbiological attributes those elated to (soil extracellular enzymes and soil basal as well as substrate induced respirations) remained relatively unexplored till date. Moreover, the comparison of effects of biochars (derived from agricultural and food wastes) with and without mineral fertilizers on crop’s photosynthetic efficiency remained neglected in the past. Therefore, we compared the effects of two types of biochars with and without mineral fertilizers on soil physico-chemical and microbiological properties and how they respond to crop growth and physiology. We considered the soil extracellular enzymes activity and microbial respiration as soil health indicators and crop’s photosystem efficiency as agronomic performance respectively for evaluating the effects of applied biochars together with mineral fertilizer. The specific objectives of the present study were to (i) compare and analyze the effects of produced biochars with and without mineral fertilizer on soil basal and substrate induced respirations and extracellular enzymes, and (ii) assess the growth and physiological responses of crop under applied amendments.

## Materials and methods

### Procurement and preparation of biochars

For the purposes of pot experiment, the food waste biochar (FWB) was prepared in two steps. The first step involved the pre-treatment process, which consists of two consequent steps i.e., the dried food waste (dry matter approx. 90%) was mixed with 25% of spruce sawdust and subsequently the mixture was pelletized at a briquetting press for the production of pellets type JGE 260 with a matrix at a size of 6 mm of extrusion holes and a pellet length of 40 mm. The second step was the heat treatment of the samples, whereby thermal pyrolysis (TP) was performed in laboratory, small-scale conditions, in a small-scale TP unit working under 600°C. This unit works discontinuously, and the maximum capacity is around 5 kg·batch^-1^ of feedstock. The glass condenser attached to the pyrolyzer was used for the separation of gaseous products and the pyrolysis oil. The input weight of feedstock samples was 3 000 g·batch^-1^. The feedstock was placed into the TP unit in a stainless-steel cylindrical reactor. During the experiments, the residence time was 340 - 410 minutes, and the temperature did not exceed 600°C.

Moreover, commercial biochar from agricultural waste was purchased from the manufacturer (Sonnenerde GmbH, Austria). This biochar was produced with a high-technology production unit Pyreg500 from grain husks, sunflower pods and pulp mud. The process temperature was set up at 650°C. The chemical composition of applied biochars is given in (**Table 1**).

**Table 1:** Chemical composition of used biochars.

	TC[%]	ROC [%]	TIC [%]	TOC [%]	N[%]	H[%]	O[%]	C:N	H:C	O:C
AB	50.13± 0.02	0.45± 0.06	0.33± 0.00	49.80± 0.02	1.01± 0.06	1.60± 0.04	17.28± 0.21	49.67± 2.89	0.03± 0.00	0.34± 0.00
FWB	81.25± 0.03	0.28± 0.01	0.07± 0.00	81.18± 0.03	3.58± 0.05	3.04± 0.06	8.10± 0.25	22.71± 0.30	0.04± 0.00	0.10± 0.00

TC, total carbon; ROC, resistant organic carbon; TIC, total inorganic carbon; TOC, total organic carbon; N, nitrogen; H, hydrogen; O, oxygen.

### Experimental design and treatments

The growth substrate used for the pot experiment was prepared by mixing a silty clay loam (USDA Textural Triangle) Haplic Luvisol (WRB soil classification) collected at field near the town Troubsko (Czech Rep., 49°10’28”N 16°29’32”E) with a fine quartz sand (0.1–1.0 mm; ≥95% SiO_2_) in a weight ratio of 1:1. The soil properties were as follows: total C 14.0 g·kg^−1^, total N 1.60 g·kg^−1^, available P 0.10 g·kg^−1^, available S 0.15 g·kg^−1^, available Ca 3.26 g·kg^−1^, available Mg 0.24 g·kg^−1^, available K 0.23 g·kg^−1^; pH (CaCl_2_) 7.3.

One kilogram of this growth substrate was mixed with 32 g (equivalent to 40 t.ha^-1^) of a particular biochar (**Table 1**) and filled to experimental plastic pots (volume 1 L, top diameter 11 cm, bottom diameter 9 cm, height 13 cm). Control treatment was left without the addition of biochar. The mineral fertilizer (MF) NPK (16:16:16) was dissolved in demineralised water and applied on soil surface of specific variants in dose equal to 0.1 g N·kg^-1^ of soil. Following biochar treatments were applied in the presence and absence of mineral fertilizer; (i) control (no biochar) (ii) foodwaste biochar (FWB) with and without mineral fertilizer (hereinafter referred to as FWB+MF) and (iii) agricultural waste derived biochar (AB) with and without mineral fertilizer (hereinafter referred to as AB+MF). The experimental treatments are shown in (**Table 2**). Each treatment was carried out in 3 replicates (pots).

**Table 2:** Description of experimental treatments.

Variant	FWB	AB	MF
Control	–	–	–
MF	–	–	✔
FWB	✔	–	–
FWB+MF	✔	–	✔
AB	–	✔	–
AB+MF	–	✔	✔

FWB, food waste derived biochar; AB, agricultural waste derived biochar; MF, mineral fertilizer. – means (not inculded) or (absent).✔ means (included) or (present).

### Pot experiment

The pot experiment with lettuce (*Lactuca sativa* L. var. *capitata*) took place in growth chamber Climacell EVO (BMT, Czech Rep.) under controlled conditions: full-spectrum LED lighting, light intensity 20,000 lux; photoperiod 12 h; temperature 18/22°C (night/day); relative humidity 70%. Lettuce seeds were sprouted on wet filter paper for two days and then five of them were sown to the depth of approximately 2 mm in each pot. After sowing, each pot was watered with 100 mL of distilled water. The 10-day-old seedlings were reduced to one plant per pot. Pot placement in the growth chamber was randomized. Soil humidity was controlled, and water content was maintained during the experiment at approximately 60% of water holding capacity. The pots were variably rotated once per week. The plants were harvested 8 weeks after sowing.

### Plant biomass and photosynthesis characteristics measurements

At harvest time, determination of photochemical efficiency of photosystem II (PSII) of lettuce plants was carried out. The quantum yield of the PSII (*Φ_PSII_*) was determined (at light intensity 2400 μmol·m^-2^·s^-1^) by the fluorometer PAR-FluorPen FP 110-LM/S (Photon Systems Instruments, Drásov, Czech Republic) and the software FluorPen 1.1 was used for the analysis of the measured data. Determination of normalized difference vegetation index (NDVI) was carried out too with PlantPen NDVI 310 (Photon System Instruments, Drásov, Czech Republic). The spectral reflectance of chlorophyll pigments, expressed as NDVI, is a measure of chlorophyll content (Garty et al., 2001) and its integrity (Castro and Sanchez-Azofeifa, 2008) and correlates with photosynthetic rate (Garty et al., 2001). Then, the lettuce shoots were cut at ground level, and the roots were gently cleaned of soil and washed with water. Fresh aboveground (AGB) and root biomass were estimated gravimetrically by weighing on the analytical scales.

### Soil analysis for microbiological soil health indicators

A mixed soil sample was taken from each pot after harvesting the lettuce. Soil samples were homogenized by sieving through a sieve with mesh size 2 mm. Air dried samples were analyzed for pH (ISO 10390, 2005) and electric conductivity (EC) (Hardie et al., 2012). Freeze-dried samples were used for the analyses of enzymatic activities: β-glucosidase (GLU), phosphatase (PHOS), urease (URE) and N-acetyl-β-D-glucosaminidase (NAG) (ISO 20130, 2018). The samples stored at 4 °C were used for determination of dehydrogenase activity (DHA) using standard method based on triphenyltetrazolium chloride (TTC) (Małachowska-Jutsz and Matyja, 2019), soil basal respiration (BR) and substrate induced respirations (IR) – D-glucose (Glc-IR), L-alanine (Ala-IR) and L-arginine (Arg-IR) (Campbell et al., 2003) using MicroResp^®^ device (The James Hutton Institute, Scotland).

### Statistical analyses

The obtained data were statistically analyzed using the one-way analysis of variance (ANOVA), Treatment means were compared using Tukey HSD *post-hoc* test (at significance level *p* = 0.05).

To evaluate the effects of applied amendments, principal component analysis (PCA) was plotted for observed variables and observations using Rstudio.

## Results

### Plant growth and chlorophyll fluorescence

**Figure 1:**
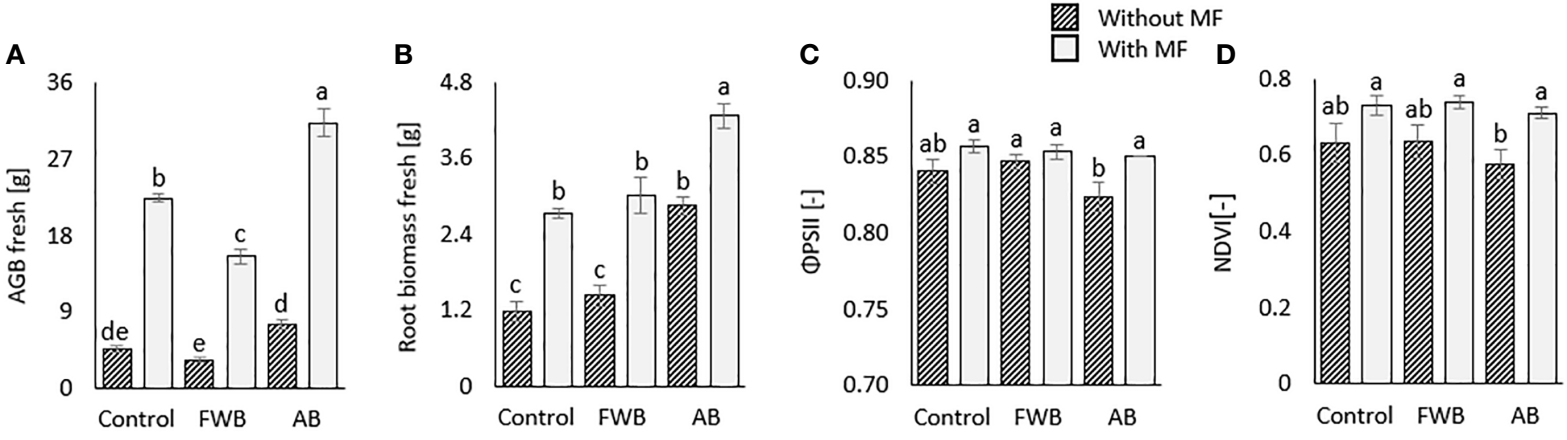
Comparative effects of applied food waste (FWB) and agricultural waste biochar (AB) with and without mineral fertilization (MF) on **(A)** above ground fresh biomass **(B)** root fresh biomass, quantum yield of the PSII **(C)** and NDVI **(D)**. Values are mean of three replicates. Different lowercase letters indicate statistical significance at p<0.05.

The application of biochars with and without mineral fertilizer (MF) differently affected the plant growth and photosynthetic parameters. The plant fresh above ground biomass (AGB-fresh) was significantly highest under AB+MF as compared to control and other treatments (**Figure 1A**). This trend was followed by MF alone and food waste biochar with mineral fertilizer (FWB+MF). The highest root fresh weight (Root-fresh) was observed under the application of AB+MF which was followed by FWB+MF relative to control (**Figure 1B**). The quantum yield of the electron transport of the PSII (Φ_PSII_), which expresses the real capacity of the PSII for photochemical reactions, was relatively increased by MF application. There was no significant increase found for Φ_PSII_, which acts as a measure of the overall efficiency of PSII reaction centers in light, under applied amendments (**Figure 1C**). The spectral reflectance of chlorophyll pigments, expressed as NDVI (**Figure 1D**) was correlated with Φ_PSII_ values (**Figure 1C**).

### Soil chemical properties

**Figure 2:**
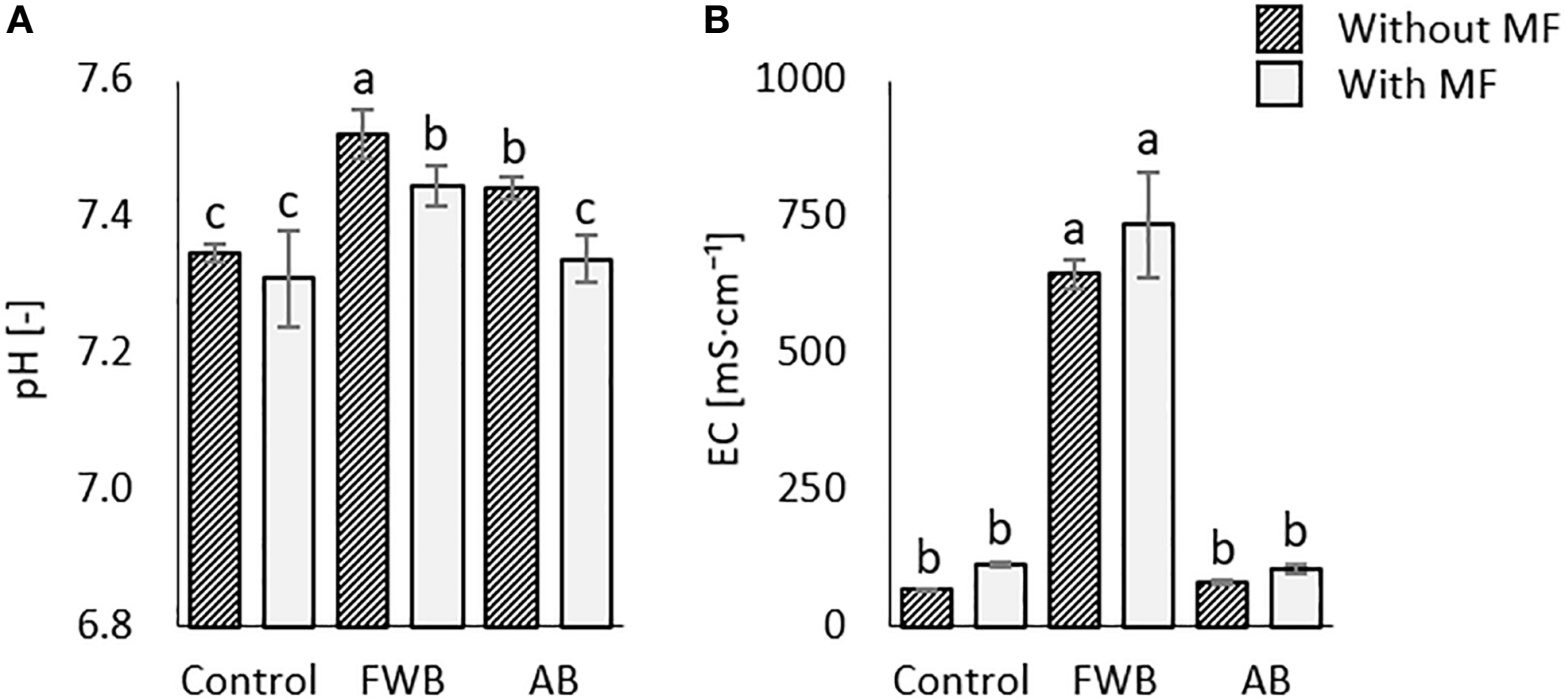
Comparative effects of applied food waste (FWB) and agricultural waste biochar (AB) with and without mineral fertilization (MF) on **(A)** soil pH and **(B)** soil electrical conductivity (EC). Values are mean of three replicates. Different lowercase letters indicate statistical significance at p<0.05.

The application of food waste biochar with and without mineral fertilization significantly enhanced the soil pH as compared to control (**Figure 2A**). The highest pH values were observed in soils receiving FWB, FWB+MF and AB, while the lowest was found under MF control which was statistically similar with the pH value under AB+MF application (**Figure 2A**). Remarkable variations were however observed for soil electrical conductivity (EC) under the applied biochars with and without MF. Specifically, the highest EC was observed under the application of FWB+MF and FWB without MF (**Figure 2B**) which were statistically significant as compared to other treatments and control.

### Soil extracellular enzymes activities

**Figure 3:**
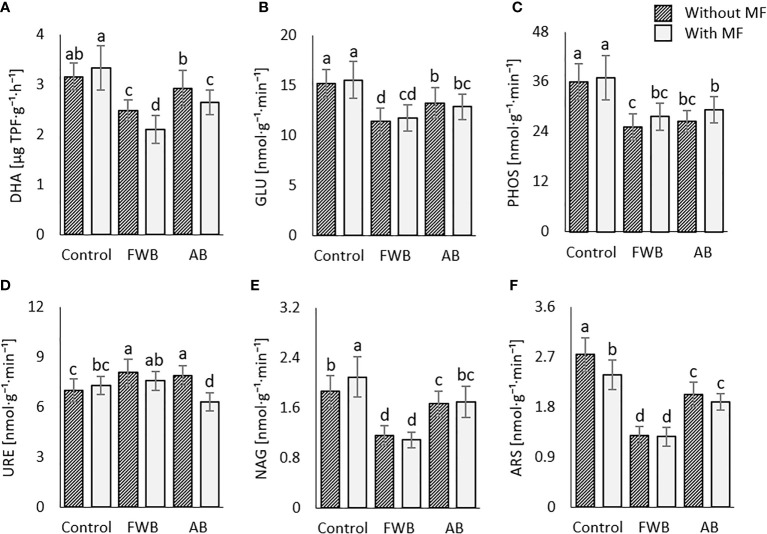
Comparative effects of applied food waste (FWB) and agricultural waste biochar (AB) with and without mineral fertilization (MF) on **(A)** dehydrogenase activity; **(B)** glucosidase activity; **(C)** phosphatase activity; **(D)** urease activity; **(E)** N-acetyl-glucosaminase activity and **(F)** aryl-sulphatase activity. Values are mean of three replicates. Different lowercase letters indicate statistical significance at p<0.05.

The highest dehydrogenase activity (DHA) was observed under the sole application of MF (**Figure 3A**). All other amendments except AB significantly reduced DHA as compared to control (**Figure 3A**). Similar to DHA, the same treatment i.e., MF resulted in highest glucosidase (Glu) and phosphatase (PHOS) activities (**Figures 3B, C**). All other amendments resulted in reduced activities of Glu and PHOS as compared to control. Regarding urease, the significantly highest activity was recorded under the application of FWB and AB without MF as compared to control (**Figure 3D**). Moreover, the MF alone enhanced N-acetyl-glucosaminase (NAG) activity as compared to other treatments (**Figure 3E**), while no clear trend was observed for aryl sulphatase activity under applied treatments (**Figure 3F**).

### Soil basal and substrate induced respiration

**Figure 4:**
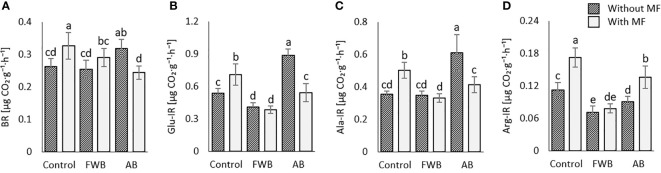
Comparative effects of applied food waste (FWB) and agricultural waste biochar (AB) with and without mineral fertilization (MF) on **(A)** soil basal respiration; **(B)** glucose-induced-respiration; **(C)** alanine-induced respiration; and **(D)** arginine-induced respiration. Values are mean of three replicates. Different lowercase letters indicate statistical significance at p<0.05.

The application of food waste and agricultural biochars with and without mineral fertilization considerably affected the basal as well as substrate induced respirations (SIR). A significantly highest increase in soil basal respiration (BR) was observed under the sole application of MF and AB (**Figure 4A**) as compared to control and other treatments. The application of AB without MF resulted in significantly highest glucose-induced respiration (Glu-IR) and alanine-induced respiration (Ala-IR) respectively as compared to control (**Figures 4B, C**). This trend was followed by the application of MF alone. However, the sole application of MF enhanced the arginine-induced respiration (Arg-IR), which was significantly highest as compared to other treatments (**Figure 4D**).

### Results from principal component analysis

**Figure 5:**
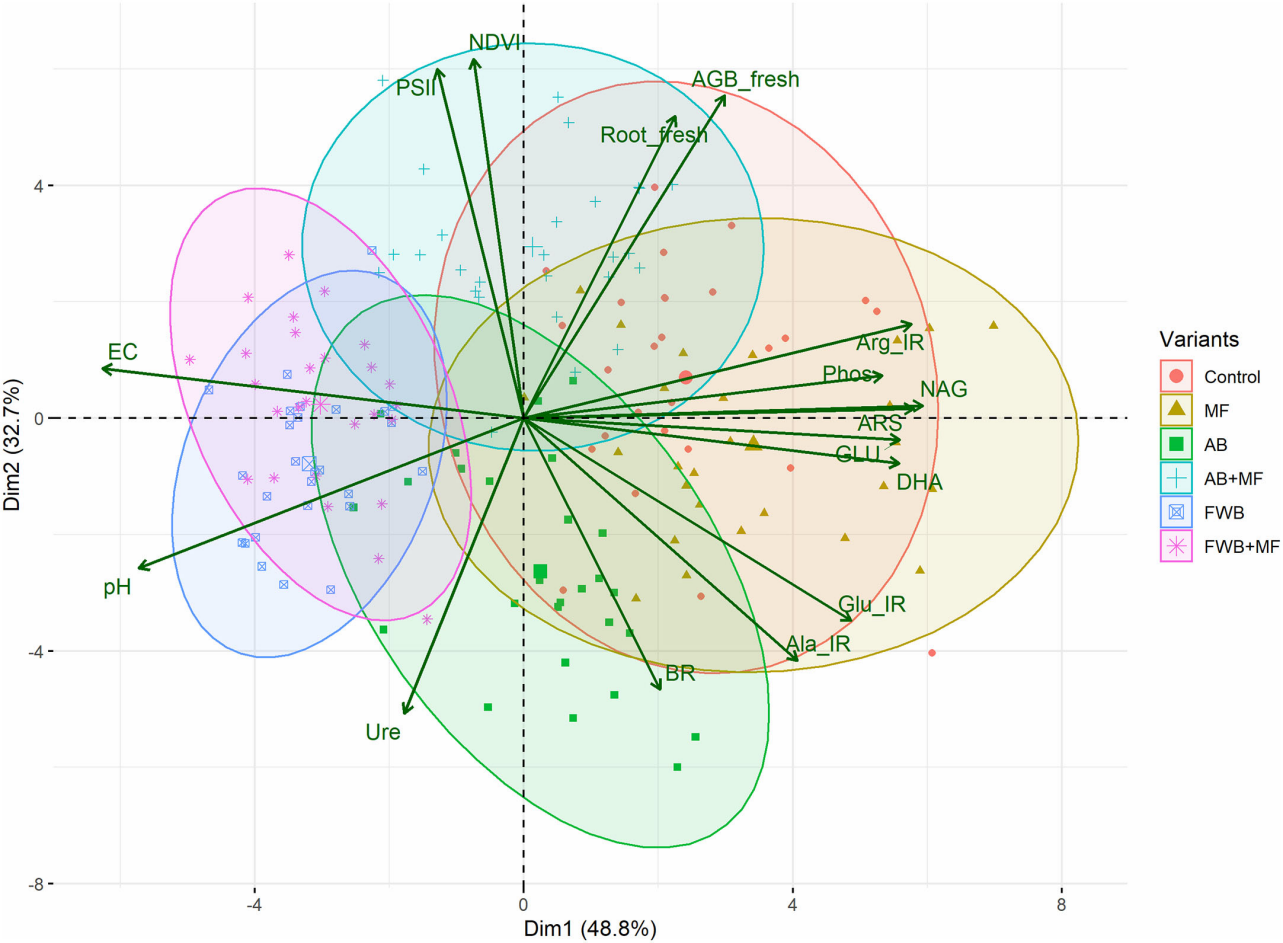
Principal component analysis of the observed microbiological and chemical soil health indicators and crop biomass and physiological parameters under the influence of applied food waste (FWB) and agricultural waste biochar (AB) with and without mineral fertilization (MF).

The score and loading plots of principal component analysis (PCA) regarding the observed soil and plant characteristics are shown in (**Figure 5**). The extracted components (Dim1 and Dim 2) maximally (82.7%) accounted for the observed variations in the data set. The applied amendments were successfully separated by the principal components (as marked by different colors). This suggests the positive influence of applied amendments on the observed parameters. The treatments MF, AB and AB+MF were distributed in components 1 and FWB and FWB+MF were distributed in component 2 of the PCA (**Figure 5**). This clearly indicated the differential roles of applied amendments on the listed soil and plant parameters. As indicated by the PC1, the applied MF was found as the most influential treatment on most of the measured soil enzymes in the present study. While PC2 showed FWB and FWB+MF as most influential treatments regarding soil chemical properties (pH and EC). The most displaced parameters were soil pH, EC and plant chlorophyll fluorescence parameters Φ_PSII_ and NDVI, suggesting the differential effects of applied amendments on soil characteristics and plant growth and physiology.

## Discussion

Agricultural and food wastes derived biochars have been regarded as alternative sources for enhancing soil chemical, physico-chemical and biological health and crop growth and development. However, the sole utilization of biochar does not always result in an increase in soil fertility and crop biomass. The present study, therefore, aimed to compare the effectiveness of agricultural and food wastes derived biochars with and without mineral fertilization for improving soil chemical, physico-chemical and biological properties related to microbial soil health indicators and crop growth. The results revealed that the application of agricultural biochar with mineral fertilization enhanced crop biomass (**Figure 1**). This enhancement might be associated with the increased acquisition of readily available plant nutrients under the applied biochar plus mineral fertilizer combination (Sadaf et al., 2017). Similarly, Jeffery et al. (2017), reported enhanced crop growth and yield. The authors stated that the enhancement of crop growth and yield is mainly related to enhanced soil nutrients under biochar application. These results are further substantiated by the findings of Lai et al. (2017) and Dong et al. (2015), who reported enhanced crop biomass and yield under applied biochar. In our work, the highest increase in biomass accumulation was observed under combined application of biochar with MF (**Figures 1A, B**), which agrees with the results of Ali et al. (2020), who reported higher crop biomass accumulation under combined application of biochar and nitrogenous fertilizers. This comparatively higher crop performance observed in the present study under AB+MF and FWB+MF revealed that integrated use of biochar and mineral fertilizers could be a suitable approach for enhancing crop production in a similar pattern observed by Singh et al. (2019a).

The higher crop growth might also be related to enhanced physiological parameters of crops under applied biochar and mineral fertilizer treatments (Qian et al., 2019; Ali et al., 2021). Despite the fact that we found no significant variations in the plant physiological parameters (ΦPSII, NDVI), their relative increase in MF fertilized treatments (**Figures 1C, D**) confirms the direct dependence of chlorophyll content and photosynthesis on nutrient availability, especially nitrogen (Huang et al., 2004; Mu and Chen, 2021). This shows that the applied biochars were unable to cast any additional benefit on crop’s physiological parameters, however, their combination with mineral fertilizers shows the potential to increase plant growth, as demonstrated also by other studies (Kizito et al., 2019; Li et al., 2020; Ndoung et al., 2021; Liu et al., 2022). The improved crop performance under combined application of biochars and mineral fertilizers could be due to the improved crop nutrient and water availability coming from fertilizer and the mechanisms of biochar on retention and exchange of these nutrients on biochar surfaces which lead consistent supply of nutrients to crops (Agbna et al., 2017; Singh et al., 2019b; Faloye et al., 2019). On the other hand, the reduced or lower crop growth performance under sole application of biochar might be due to the clogging of micropores and reduced availability of crop nutrients (Singh et al., 2019b).

Soil chemical and physico-chemical properties are important determinants of soil quality. It has been shown that biochar application results in the modification of soil chemical properties mainly pH and electrical conductivity and soil nutrient status (Joseph et al., 2020; Holatko et al., 2022). We found enhanced soil pH under the application of food waste biochar (FWB) with and without MF (**Figure 2A**). This enhanced soil pH under biochar addition is related to the higher pH of the biochar itself and its liming effect as has been previously reported by many researchers (Ali et al., 2020; Hammerschmiedt et al., 2022). Moreover, the highest increase in EC under FWB+MF treatment than control (**Figure 2B**) might be the outcome of direct release of nutrients from MF which could be retained on the biochar surfaces and resulted in increased soluble salts in soil solution eventually showing higher EC. This is further supported by the results of PCA (**Figure 5**) suggesting FWB with and without MF as most influential treatments for soil chemical properties observed here. Moreover, higher pH and EC might be due to the higher porosity and surface area of biochar which together with applied fertilizers might have improved the soil physico-chemical properties through nutrient retention on biochar surfaces resulting in higher pH and EC (Jaafar et al., 2015). Our results are in line with Ali et al. (2021) who reported enhanced soil physico-chemical properties due to the application of biochar and N fertilizers.

Soil extracellular enzymes mediate the cycling of C, N and P in agroecosystems and are important determinants of soil organic matter decomposition (Bilen and Turan, 2022). We found differential responses of applied organic amendments with and without MF on various soil enzymes involved in C, N and P cycling (**Figures 3A–F**). In most of the cases, application of MF enhanced soil enzyme activities. Our findings agree with Tian et al. (2016) reporting enhanced soil enzyme activities under the application of mineral fertilization. Both mineral fertilization and biochar have been recognized to improve soil extracellular enzyme activities. The enhancement of enzyme activities under MF in the present study could be related to the increased availability of limiting nutrients to microbes as speculated by Zhang et al. (2014). Moreover, it has been acknowledged that the application of mineral fertilizers causes rapid mineralization of native soil organic matter (Foley et al., 2005; Liu et al., 2010), which is reflective in the findings obtained on enhanced activity of nutrient mineralizing enzymes under MF application in the present study (**Figure 3**). Moreover, Lehman et al. (2011) postulated that the alterations of soil pH due to biochar addition might affect the activities of enzymes especially phosphatases. Furthermore, in line with our findings, Song et al. (2020) in another study reported enhanced activity of C and N acquiring enzymes under the influence of mineral fertilization and biochar additions (**Figure 3**). Thus, the higher enzyme activities under applied amendments are suggestive for increased nutrients (C, N, P) mineralization in this study.

Soil respiration is one of the biological soil health indicators and is of significant concern in the face of climate change (Singh et al., 2019b). Considerable variations were observed for soil basal, and substrate induced respirations in soils subjected to various amendments (**Figures 4A–D**). The soil basal and SIR are considered active indicators of soil microbial biomass (Hassink, 1993). The sole application of MF yielded highest BR and Arg-IR while the amendment with AB without MF enhanced Glu-IR and Ala-IR in the present study (**Figures 4A–C**). We ascribe the higher BR and Arg-IR to increased utilization of nutrient sources by microbes and their proliferation under the application of MF and arginine (substrate). Moreover, the role of biochar in improving SIR has been well studied in many studies (Gul et al., 2015; Karimi et al., 2020). The enhanced SIR under AB in the present study might be related to an enhanced substrate availability and release of other biologically active compounds (Hermann et al., 2019). Moreover, biochar porosity provides the microbes with essential microenvironment, water and aeration, thereby enhancing their activity (Gul et al., 2015; Xu et al., 2016; Hermann et al., 2019). Generally, higher soil respiration is observed in biochar treated soils which further gets increased or decreased depending on biochar types and the amount of labile carbon present (Jones et al., 2012; Brunn et al., 2014; Mohan et al., 2018). The increase in Glu-IR and Ala-IR in the present study under applied AB treatment might reveal higher microbial activity due to the presence of more labile C (Hussain et al., 2017). Moreover, the higher variations observed for soil enzymes (**Figure 3**) and soil respiration (**Figure 4**) under applied amendments might be associated with the large differences in C:N ratios of applied biochars (**Table 1**). This could have caused large variations in microbial growth and nutrient turnover and hence caused variations on observed microbial attributes.

## Conclusion

Comparison and analysis of food and agricultural wastes derived biochar in combination/absence of mineral fertilizer revealed differential responses of soil microbial indicators and plant growth and physiological alteration. The study demonstrated that agricultural waste derived biochar enhanced crop growth and its combination with mineral fertilizers had the potential to improve its physiological attributes in terms of chlorophyll fluorescence indicators. This shows that the integration of biochar with mineral fertilizers could be a sustainable approach for enhancing crop production. The food waste derived biochar on the other hand, was found to enhance soil chemical properties owing to its alkaline nature and higher nutrient contents. Furthermore, the mineral fertilizer was most influential strategy in improving soil basal respiration and C, N and P cycling enzymes which suggests the role of fertilizers in nutrient cycling as indicated by the principal component analysis as well. It is thus concluded that, the application of food and agricultural waste derived biochars not only helps in waste recycling but also help in modification of soil bio-chemical properties together with mineral fertilizers. Based on findings, it can be concluded that a combination of biochar and mineral fertilizers is a viable approach for sustainable soil management and crop production in agro-ecosystems. However, further studies taking into account the functional groups characterization and surface chemistry of biochars derived from various wastes are required to deepen our understanding on the mechanisms by which biochar affects soil quality attributes and improve crop performance.

## Data availability statement

The original contributions presented in the study are included in the article/supplementary material. Further inquiries can be directed to the corresponding authors.

## Author contributions

AM, JH and MB: conceptualization. MB, TH, AK, OM: methodology. TH, TC, and TB: software. MN, JK, AK and JH: validation. MB and PS, TH: formal analysis. TC, AK, TB and OM: resources. TB and OM: data curation. AM: writing—original draft preparation. JK, AM, MB, TH, PS, MN and JH: writing— review and editing. MB, JK and TC: supervision. MB, JK, AK, TC and JH: project administration. MB, JK, AK and JH: funding acquisition. All authors have read and agreed to the published version of the manuscript.

## Funding

The work was supported by the projects of Technology Agency of the Czech Republic TJ02000262 and TH03030319 and by the Ministry of Agriculture of the Czech Republic, institutional support MZE-RO1218, MZE-RO1722 and by Ministry of Education, Youth and Sports of the Czech Republic, grant number FCH-S-22-8001.

## Conflict of interest

AK was employed by the company Agricultural Research, Ltd. and JH was employed by the company Agrovyzkum Rapotin, Ltd.

The remaining authors declare that the research was conducted in the absence of any commercial or financial relationships that could be construed as a potential conflict of interest.

## Publisher’s note

All claims expressed in this article are solely those of the authors and do not necessarily represent those of their affiliated organizations, or those of the publisher, the editors and the reviewers. Any product that may be evaluated in this article, or claim that may be made by its manufacturer, is not guaranteed or endorsed by the publisher.
